# Heated humidified high-flow nasal cannula: a new conservative approach for neonatal nasal stenosis

**DOI:** 10.1007/s00405-024-08728-4

**Published:** 2024-05-14

**Authors:** Shany Havazelet, Patrick Stafler, Ihab Zarzur, Tara Coreanu, Roy Hod, Keren Armoni-Domany, Dror Gilony

**Affiliations:** 1 Department of Otolaryngology - Head and Neck Surgery, Rabin Medical Center, Petach Tikva, Israel; 2 Institute of Pediatric Pulmonology, Schneider Children’s Medical Center of Israel, Petach Tikva, Israel; 3 Department of Pediatric Otolaryngology, Schneider Children’s Medical Center of Israel, Petach Tikva, Israel; 4 Faculty of Medicine, Tel Aviv University, Tel Aviv, Israel; 5 Department of Pediatrics, Edith Wolfson Medical Center, Holon, Israel

**Keywords:** Heated humidified high-flow nasal cannula (HFNC), Nasal stenosis, Congenital pyriform aperture stenosis, Sinonasal surgical treatment, Pediatric respiratory management

## Abstract

**Purpose:**

The aim of this study is to evaluate the efficacy of heated humidified high flow nasal cannula (HFNC) therapy as a conservative treatment option for newborns suffering from nasal stenosis, a condition that often leads to respiratory distress and feeding difficulties. Given the increasing utilization of HFNC in various upper and lower respiratory tract indications, characterized by its flow-based mechanism and minimal mucosal damage, we seek to investigate its potential benefits in this specific patient population.

**Methods:**

A retrospective chart review of newborns with congenital nasal stenosis treated with HFNC for respiratory distress or feeding difficulties in a pediatric tertiary center between 2014 and 2022. Data were collected for demographic characteristics, clinical presentation and ventilatory requirements, pre and post HFNC application.

**Results:**

Six infants with nasal stenosis were included in the study cohort. Five were diagnosed with congenital pyriform aperture stenosis, three of whom had additional midnasal stenosis. One patient had nasal synechiae. Two patients had failed surgical treatment and all patients failed conservative treatment prior to HFNC treatment. Following HFNC use, improvement was noted in oxygen saturations, heart and respiratory rates, meal volumes and weight. None of the patients required any additional sinonasal surgical treatment. No complications were observed.

**Conclusions:**

In this case series, we present the first documented use of HFNC treatment for nasal stenosis, showing favorable results. Further studies with a larger cohort, wider range of conditions and extended follow-up periods are needed to establish the risks and benefits of HFNC for neonatal nasal stenosis.

## Introduction

Congenital nasal stenosis poses significant challenges for newborns, as their obligatory nasal breathing predisposes them to shortness of breath and feeding difficulties [1]. Furthermore, iatrogenic nasal stenosis can arise from repeated nasal trauma, including nasogastric tube insertions and repeated nasal suctioning, particularly in cases of premature infants in intensive care settings [2]. Severe stenosis may lead to extreme respiratory distress, feeding challenges, and failure to gain weight [3]. The primary approach to management is conservative, aiming to preserve sufficient airway patency. This involves the use of topical decongestants, saline irrigations, humidifiers, or nasal suction [4]. Severe cases may warrant continuous oxygen support or even require mechanical ventilation to alleviate hypoxia.

Despite initial attempts at conservative management, inadequate improvement may require resorting to a surgical intervention focused on expanding the nasal aperture. Nevertheless, surgical management has emerged as the definitive treatment modality in more than 80% of nasal stenosis cases [5]. Consequently, the pivotal role of the otolaryngologist in diagnosing and selecting the optimal surgical strategy for addressing nasal stenosis cannot be overstated.

Surgical interventions for congenital nasal stenosis are performed within a limited anatomical space. This may contribute to complications such as synechia, restenosis or injuries to the lacrimal duct and tooth buds [6]. Consequently, surgical management poses a considerable risk of long-term complications, warranting non-interventional alternatives whenever feasible.

Heated humidified high flow nasal cannula (HFNC) treatment has become a valuable tool in managing respiratory insufficiency in children, resulting from parenchymal or airway etiologies. This modality administers a humidified air-oxygen mixture through nasal prongs, ventilating both the upper and lower respiratory tracts, while minimizing damage to the mucous membranes [7].

HFNC demonstrated success in treating respiratory distress syndrome in preterm infants [8]. Further investigations have explored its potential in managing obstructive sleep apnea (OSA) in children, yielding favorable outcomes comparable to CPAP treatment [9, 10]. Initially used within the hospital setting, treatment at home is now supported for a variety of indications [11].

In the present study, we present a case series examining a novel application of HFNC in the context of conservative treatment for newborns with nasal stenosis. This innovative approach aims to improve respiratory function and alleviate feeding difficulties, offering a potential alternative to surgical interventions for this vulnerable patient population.

## Methods

### Study design and setting

This was a retrospective single center cohort study, examining demographic and clinical data of children aged 0–18 years with congenital nasal stenosis who were treated with HFNC for respiratory distress and/or failure to gain weight between the years 2014–2022 at Schneider Children's Medical Center of Israel, a tertiary pediatric center.

Included in the study were children diagnosed with congenital nasal stenosis, as confirmed by physical examination including Fiberoptic Rhinolaryngoscopy, who displayed persistent respiratory distress despite conservative measures. Computed tomography (CT) imaging complemented the diagnosis, aiding in cases where the scope exam could not be complete due to severe stenosis. Assessments of stenotic area were conducted in the axial view, with the image aligned parallel to the plane of the bony palate using multi-plane reconstruction techniques. Measurements of stenosis dimensions and locations, including mid-nasal, pyriform aperture, and choana, were performed according to the method outlined by Levi et al. [12].

Medical records were reviewed for demographic characteristics and clinical data prior and subsequent to HFNC use, focusing on respiratory and growth status. All oxygen saturation, respiratory and heart rate measurements recorded within 48 h pre and 48 h post commencing HFNC were collected and averaged. Patients’ feeding volumes per meal, averaged over 24 h periods prior to commencing HFNC and one month post start of treatment were recorded, as was weight, using the closest available measurements prior to and one month post start of treatment. Weight growth percentage was calculated using the WHO child and toddler growth chart [13]. Technical data on HFNC use were collected including fraction of inspired oxygen (FiO_2_), flow rate, temperature, and humidity.

### Treatment protocol

Children identified as eligible for HFNC treatment were trialed on the Precision flow (Vapotherm) during an inpatient stay. They were monitored clinically and with a pulse oximeter to determine benefit in terms of respiratory distress and ability to feed. Blood gases were not measured routinely. Air flow was titrated as tolerated, usually aiming to achieve a flow rate of 2 L/kg/min. Once children were found to benefit from HFNC, a home device was ordered. A humidifier and flow generator device (myAirvo 2; Fisher & Paykel Healthcare) were used to deliver high flow via nasal cannulae. These were chosen according to nasal aperture and weight, to achieve optimal flow rate (Optiflow Junior S or M; Fisher & Paykel Healthcare). The nasal cannula maintained a temperature of 34°C and relative humidity of 100% at the nasal outlet as reported by the manufacturer.

Children were required to remain inpatients for at least one further night using the home device, to adapt the interface and ensure safety and effective ventilation. Parents were trained in the use of the device and underwent a resuscitation tutorial. Patients were prescribed a home pulse oximeter and, when required, suction device and oxygen concentrator. A home ventilation team was assembled, consisting of a technician and a physician, tasked with regular home visits, maintenance, and trouble shooting.

### Statistical analysis

Statistical analyses were performed with SPSS software, version 25.0 (Armonk, NY: IBM Corp.). Continuous variables were described using median and range, categorical variables were described using percentage.

## Results

### Patient characteristics

The study cohort initially included seven children (six males and one female) with median gestational age of 39 weeks (range 25–40). Notably, one patient with VACTERL syndrome was diagnosed with congenital multi-level nasal stenosis through CT imaging. This patient commenced HFNC therapy at 21 weeks but struggled to adapt to the device, ultimately requiring a tracheostomy at a different medical facility less than a month after initiating HFNC treatment, where he continued follow-up care.

For the final cohort, congenital nasal stenosis was diagnosed at a median age of 3 weeks (Range 2–7). Most of these children had additional comorbidities resulting from developmental congenital anomalies, such as central line anomalies syndromes. The location of nasal stenosis varied among patients, encompassing pyriform aperture, mid-nasal, and multilevel stenosis. Median follow-up time was 14 months (Range 6–46). Demographic and clinical characteristics of the participants are provided in Table 1*.*

**Table 1:** Patient characteristics

Patient #	Gestational age at birth (weeks)	Gender	Weight at birth (Kg)	Age at diagnosis (weeks)	Comorbidities	Feeding route	Type of stenosis	Additional nasal pathology	Treatment before HFNC
1	39	Male	3.3	3	–	PO	CPAS	Septal deviation	Dethamycin nasal drops
2	39	Male	2.8	3	Adrenal insufficiency, inner ear anomaly	PO	CPAS	Septal deviation	Dethamycin nasal drops
3	38	Male	2.4	2	Hypoplastic heart	PEG	CPAS, midnasal	Septal deviation	Dethamycin nasal drops
4	25	Male	1	7	BPD, retinopathy	PO	Synechia, choanal	–	ESS for adhesionlysis
5	39	Male	3.9	2	–	PO	CPAS	–	Saline drops, oral steroids
6	40	Female	3.4	4	Central line anomalies	PEG	CPAS, choanal	–	Surgical turbinate deviation and stenosis expansion

CPAS: congenital pyriform aperture stenosis; BPD: bronchopulmonary dysplasia; ESS: Endoscopic sinus surge

### Clinical presentation

All patients exhibited symptoms indicative of upper airway obstruction, leading to respiratory distress. In five of them, feeding difficulties were associated, with two requiring feeding through percutaneous endoscopic gastrostomy (PEG) to ensure sufficient intake.

Three patients underwent Computer tomography (CT) prior to initiating HFNC therapy, to reveal the level of the stenosis. All received conservative treatment including dethamycin nasal drops. One patient received saline nasal drops combined with oral steroids. Two patients underwent surgical procedures. The surgical interventions included turbinate deviation and stenosis expansion in one case, and lysis of adhesions in another. These treatments yielded insufficient improvement with subsequent implementation of HFNC therapy.

### HFNC usage

The median initiation age for HFNC usage was recorded at 12.5 weeks, encompassing a wide range from 3 to 68 weeks. Mean age at initiation was 22 weeks (SD 23.7). This variability is mainly owing to one patient who was referred to our medical center following an unsuccessful prior surgical intervention elsewhere.

There was also notable heterogeneity with regards to the duration of HFNC utilization, spanning from a brief one-month period of usage to continuous application by 13 months of follow-up. The collective median duration of HFNC use amounted to 9 months (range 1–15). Whilst all patients used the device during sleep, four patients were reliant on it during the day as well. Instances of truncated use predominantly stemmed from difficulties attaining optimal fit of the cannula.

Individualized HFNC device configurations were tailored to patient attributes and demands. Further details about HFNC treatment are shown in Table 2*.*

**Table 2:** HFNC Usage Summary

Patient #	Age at HFNC initiation (weeks)	HFNC flow (L/kg/min)	% Fractional inspired Oxygen	Type of use	Duration of myAirvo use (months)
1	3	2	25	24/7	15
2	3	2	30	Sleep	8
3	38	2	21	24/7	13*
4	20	0.5	24	Sleep	7
5	5	2	21	Night ± day	12
6	68	1	21	24/7	1

^*^Continuous use, exceeding follow-up period

### Treatment outcomes

Table 3 shows the impact of HFNC institution on a number of variables. Increased oxygen saturation, with or without oxygen supplementation, was observed in all individuals commenced on HFNC therapy. Furthermore, mean respiratory and heart rates tended to drop following institution of HFNC, indicating physiological benefit through reduced work of breathing.

**Table 3:** Treatment outcomes

Patient #	Mean O_2_ Saturation (%)^a^		Mean RR^a^		Mean HR^a^		Feeding volume per meal (ml)^b^		Weight Growth Centile^c^	
	Pre-treatment (room air)	Post -treatment	Pre-treatment	Post-treatment	Pre-treatment	Post-treatment	Pre-treatment	Post-treatment	Pre-treatment (%)	1-month post-treatment (%)
**1**	60	100^d^	50	N/A	180	138	60	90	0	41
**2**	89	97^d^	60	N/A	180	140	90	120	9.7	26.1
**3**	72	80	27	20	122	110	100	140	0	41
**4**	N/A	N/A	N/A	55	N/A	N/A	120	180	40	60
**5**	85	100	42	30	177	136	N/A	N/A	10	68.9
**6**	82	98	N/A	32	130	122	N/A	145	0.1	1.8

RR: respiratory rate; HR: heart rate

^a^Mean of all measurements recorded 48 h pre and 48 h post commencing HFNC

^b^Patients’ feeding volumes per meal, averaged over 24-h periods prior to commencing HFNC and one month post start of treatment

^c^According to the WHO Child Toddler Growth Chart(11)

^d^O_2_ supplementation (see details in Table 2)

Notably, feeding routines were observed to improve in all four orally fed patients with increase intake volumes compared to those tolerated prior to the commencement of HFNC therapy. After one month of treatment, assessments of growth centiles showed improved weight gain in all individuals.

## Discussion

The incidence of nasal stenosis, though infrequent, poses significant risks for serious medical complications, notably impacting respiration and feeding [3, 14]. To our knowledge this is the first study to suggest the use of heated humidified high-flow nasal cannula (HFNC) as a conservative solution for the treatment of nasal stenosis. Our preliminary study demonstrated encouraging results in improving respiratory and feeding difficulties.

Navigating the course of nasal stenosis management hinges fundamentally on the interplay of symptom severity and the patient's clinical trajectory. Mild cases can be effectively managed with conservative interventions like short-term intranasal corticosteroids or nasal decongestants, whereas moderate to severe obstruction scenarios necessitate surgical interventions [14]. In a study by Chakravarty et al. 84% of nasal stenosis cases necessitated surgical intervention after inadequate improvement despite medical management [5]. Hence, it is imperative for otolaryngologists to thoroughly explore conservative treatment options before considering surgical intervention as the next step.

Surgical approaches described in literature include removal of scar tissue, replacement with graft tissue, and post procedure stenting to reduce restenosis. Over time, however, these surgical techniques have seen limited transformative evolution, with only a handful of innovative alternatives proposed. For instance, Adams et al. suggested use of steel gauging earrings for dilation and stenting for the treatment of nasal stenosis [15]. Nasal stenosis presents intricate challenges for surgeons, mainly attributed to the inherent lack of cartilaginous structural support in the ala, a susceptibility to scar contracture, and an increased risk of restenosis [16].

Considering the transient nature of neonatal nasal stenosis, a conservative therapeutic approach is considered preferable as the primary line of management. In a noteworthy case series, Karplus et al. demonstrated the efficacy of conservative measures in managing nasal stenosis in five neonates. The approach encompassed nasal drops, frequent nasal suction, and temporary nasopharyngeal intubation, effectively ameliorating symptoms and negating the necessity for surgical intervention [17]. In addition, Kemal et al. described the use of a nasal trumpet as a non-invasive treatment method in congenital nasal stenosis, removed 1.5 months later with no further need of use [12, 18].

The most prevalent modalities used for noninvasive respiratory support are nasal continuous positive airway pressure (NCPAP) and heated humidified high-flow nasal cannula (HFNC). In cases of pediatric obstructive sleep apnea, both have demonstrated comparable therapeutic effect among children with obesity and medical complexities, yielding similar reductions in polysomnography quantified measures of OSA severity [9]. However, HFNC showed promise as an alternative for children with OSA who struggle with CPAP adherence, particularly in cases where CPAP usage is refused due to inadequately fitting masks or other practical constraints [19]. Furthermore, a meta-analysis conducted by Lou et al. highlighted the advantages of HFNC over NCPAP in terms of enhancing feeding tolerance [20].

Our therapeutic approach is founded on the understanding that nasal stenosis represents a transient condition that tends to improve as the patient goes through natural growth and development. Consequently, surgical interventions, which bear the potential for enduring lasting complications, are an unfavorable course of action. Employing HFNC support until the nasal stenosis resolves offers a comfortable and non-permanent solution, mostly enabling patients to maintain their regular daily activities.

It is imperative to acknowledge the inherent limitations of our study, primarily stemming from its small sample size and retrospective design. Furthermore, the lack of a formal definition or classification system for nasal stenosis introduces potential variability in diagnosis interpretation; although we utilized both clinical and imaging data to define the diagnosis, other studies may adopt different criteria, emphasizing the need for a standardized definition. Moreover, considering the retrospective nature of the study, obtaining objective measurements from case notes at the time of admission was not always feasible. Consequently, further studies, with a larger number of participants and a wider range of indications, are warranted to establish the utility of HFNC in the context of neonatal nasal stenosis accompanied by respiratory distress, as well as to evaluate the long-term effects of this novel therapeutic approach.

## Conclusions

This case series underscores the potential of HFNC as a novel approach to conservatively manage respiratory distress from nasal stenosis, particularly in scenarios where conventional conservative or surgical strategies are deemed inadequate. The present study sets the stage for future research into the efficacy of HFNC within this specific patient population.
